# Combination of CTLA-4 and PD-1 blockers for treatment of cancer

**DOI:** 10.1186/s13046-019-1259-z

**Published:** 2019-06-13

**Authors:** Anand Rotte

**Affiliations:** Clinical & Regulatory Affairs, Nevro Corp, 1800 Bridge Parkway, Redwood City, CA 94065 USA

**Keywords:** Immunotherapy, CTLA-4, PD-1, Combination therapy

## Abstract

Targeting checkpoints of immune cell activation has been demonstrated to be the most effective approach for activation of anti-tumor immune responses. Cytotoxic T-lymphocyte-associated protein 4 (CTLA-4) and programmed cell death protein 1 (PD-1), both inhibitory checkpoints commonly seen on activated T-cells have been found to be the most reliable targets for the treatment of cancer. Six drugs targeting PD-1 or its ligand PD-L1 and one drug targeting CTLA-4 have been approved for treatment of different types of cancers and several others are in advanced stages of development. The drugs when administered as monotherapy had dramatic increase in durable response rates and had manageable safety profile, but more than 50% of patients failed to respond to treatment. Combination of CTLA-4 and PD-1 blockers was then evaluated to increase the response rates in patients, and ipilimumab (anti-CTLA-4) plus nivolumab (anti-PD-1) combination was shown to significantly enhance efficacy in metastatic melanoma patients. Subsequently, ipilimumab plus nivolumab was approved for treatment of metastatic melanoma, advanced renal cell carcinoma and metastatic colorectal cancer with MMR/MSI-H aberrations. The success of combination encouraged multiple clinical studies in other cancer types. Efficacy of the combination has been shown in a number of published studies and is under evaluation in multiple ongoing studies. This review aims to support future research in combination immunotherapy by discussing the basic details of CTLA-4 and PD-1 pathways and the results from clinical studies that evaluated combination of CTLA-4 and PD-1/PD-L1 blockers.

**Electronic supplementary material:**

The online version of this article (10.1186/s13046-019-1259-z) contains supplementary material, which is available to authorized users.

## Background

For several decades treatment of advanced cancer has been challenged by lack of reliable therapeutic options. Patients with metastatic tumors that were not surgically resectable had to depend on chemotherapy, which is commonly associated with severe adverse events as well as high rates of relapse. As the understanding of immune system and immune surveillance grew, the idea of utilizing immune cells to eliminate cancer gained significance and various strategies to activate immune response were developed. Administration of interleukin-2 (IL-2), a cytokine known for stimulating T-cell proliferation, is one of the earliest approach tested for cancer treatment and IL-2 is one of the oldest immune based drug approved for the treatment of cancer [1–3]. However, the first generation of immunotherapies were limited by low response rates and high incidence of serious adverse events [4]. The search for dependable targets for the modulation of immune responses led to the discovery of checkpoints of T-cell activation and development of monoclonal antibodies targeting the checkpoints [5–11]. Among the checkpoints, cytotoxic T-lymphocyte-associated protein 4 (CTLA-4) and programmed cell death protein 1 (PD-1) have been found to be the most reliable targets and drugs targeting CTLA-4 and PD-1 drastically changed the outcomes of treatment for advanced cancers. To date, 7 drugs targeting CTLA-4/PD-1 are approved for treatment of different types of cancers including melanoma, lung cancer, breast cancer, head and neck cancer, bladder cancer, Merkel cell cancer, cervical cancer, hepatocellular cancer, gastric cancer, cutaneous squamous cell cancer, classic Hodgkin’s lymphoma and B-cell lymphoma (Table 1). The impact of CTLA-4 and PD-1 blockers on cancer research and their success in cancer treatment is acknowledged by researchers as well as clinicians worldwide and rightfully the Nobel Prize in Physiology or Medicine for 2018 was awarded to Professor James Allison, MD Anderson Cancer Center, USA and Professor Tasuku Honjo, Kyoto University, Japan for their research on CTLA-4 and PD-1 respectively [12].

**Table 1:** List of approved drugs targeting CTLA-4 and PD-1 (current as May 2019)

Drug	Brand name	Indication
CTLA-4 blockers		
Ipilimumab	Yervoy	As monotherapy for metastatic melanoma and surgically resectable ‘high-risk’ melanoma (adjuvant setting)
PD-1 blockers		
Nivolumab	Opdivo	Metastatic melanoma, metastatic non-small-cell lung cancer (NSCLC), renal cell carcinoma (RCC), classical Hodgkin’s lymphoma, head and neck squamous cell carcinoma (HNSCC), metastatic urothelial carcinoma, hepatocellular carcinoma (HCC), colorectal cancer with MSI-H and MMR aberrations
Pembrolizumab	Keytruda	Metastatic melanoma, surgically resectable ‘high-risk melanoma (adjuvant setting), metastatic NSCLC, classical Hodgkin’s lymphoma, primary mediastinal B-cell lymphoma (PMBCL), HNSCC, gastric cancer, solid tumors with MSI-H and MMR aberrations, metastatic urothelial carcinoma, Merkel cell carcinoma, renal cell carcinoma, cervical cancer, hepatocellular carcinoma,
Cemiplimab	Libtayo	Metastatic cutaneous squamous cell carcinoma (CSCC) or locally advanced CSCC who are not candidates for curative surgery or curative radiation
PD-L1 blockers		
Atezolizumab	Tecentriq	Metastatic urothelial carcinoma, metastatic NSCLC (monotherapy and in combination with chemotherapy), metastatic SCLC (in combination with chemotherapy) and metastatic triple negative breast cancer (in combination with paclitaxel)
Avelumab	Bevencio	Merkel cell carcinoma, metastatic urothelial carcinoma
Durvalumab	Imfinzi	Metastatic urothelial carcinoma, unresectable stage III NSCLC
Combination of CTLA-4 and PD-1 blockers		
Ipilimumab plus nivolumab	Yervoy plus Opdivo	Metastatic melanoma, metastatic renal cell carcinoma, colorectal cancer with MSI-H and MMR aberrations

Main advantages of CTLA-4 and PD-1 blockers are impressive durable response rates and manageable adverse events, but only a fraction of patients were seen to respond to monotherapy [13–15]. Combination of CTLA-4 and PD-1 blockers was suggested to have synergistic effect on activation of anti-tumor immune response and to increase the response rates in patients. Multiple clinical studies were conducted to test the safety and efficacy of the combination in different cancer subtypes. The combination showed remarkable increase in response rates and median survival times in melanoma and renal cell carcinoma, resulting in approval of the ipilimumab and nivolumab combination for their treatment. Additional studies in difficult to treat cancer types such as non-small cell lung cancer, mesothelioma, sarcoma and esophagogastric cancers have shown improved response rates in patients treated with combination therapy. The present review aims to discuss the results from clinical studies that evaluated combination of CTLA-4 and PD-1 blockers to support future research in combination immunotherapy. Basic details of CTLA-4 and PD-1 including their expression, ligands and role in immune response are described in the following sections to help in easier understanding of mechanisms of action.

### CTLA-4

CTLA-4 (cluster of differentiation 152, CD152), is a receptor found on surface of activated T-cells. It was discovered through screening of mouse cytolytic T-cell derived cDNA libraries by Brunet et al in 1987 [16]. The location of human CTLA-4 gene and the details of the protein encoded by CTLA-4 gene are listed in Table 2. CTLA-4 expression is normally seen upon activation of T-cells, but regulatory T-cells (Tregs), express CTLA-4 constitutively due to their high levels of forkhead transcription factor FoxP3, which is known to regulate CTLA-4 expression [17–19]. CTLA-4 mainly acts by competing with CD28 receptors for binding to B7 ligands (B7-1/CD80 and B7-2/CD86) on antigen presenting cells (APCs). During T-cell activation, CD28 receptors on T-cells bind to B7 ligands on APCs and provide the essential second activation signal for T-cells. However, CTLA-4 receptors bind to B7 ligands with higher affinity and at a lower surface density and thereby outcompete CD28 receptors for binding with B7 ligands. Lack of second activation signal in presence of CTLA-4 receptors would thus lead to anergy in T-cells [20–22]. In addition, CTLA-4 receptors are also shown to sequester B7-ligands from the surface of the APCs and result in significant depletion of the ligands on their surface.

**Table 2:** Summary of CTLA-4 and PD1

Receptor	CTLA-4	PD-1
Synonyms	CD152	PDCD1, CD279
Gene location	Chromosome 2q33	Chromosome 2q37.3
Protein details	Amino acids #223 Type 1 transmembrane glycoprotein belonging to Ig super family Dimer Domains: a single peptide, an extracellular ligand-binding domain, a transmembrane domain, and a short cytoplasmic tail	Amino acids #288 Type I transmembrane protein belonging to Ig super family Monomer Domains: extracellular N-terminal IgV-like domain, a transmembrane domain, and a cytoplasmic tail
Signaling motif	Cytoplasmic tail	ITSM
Cells expressing receptor	Effector T-cells & TRegs	Effector T-cells, TRegs, NK cells & macrophages
Ligands	CD80 (B7-1), CD86 (B7-2)	PD-L1 (B7-H1), PD-L2 (B7-DC)
Cells expressing ligands	APCs	APCs, hematopoetic & nonhematopoetic cells & tumor cells

Intriguingly, due to its structural similarity with CD28 and its expression on activated T-cells, CTLA-4 was thought to be a positive regulator of T-cells in the initial days of its discovery. Professor Allison is credited for demonstrating the negative role of CTLA-4 and establishing the opposing effects of CTLA-4 and CD28 in response to T-cell stimulation. His research clearly showed that CTLA-4 engagement with B7-ligands abrogated IL-2 secretion by T-cells and T-cell proliferation that followed TCR activation; that blockade of CTLA-4 using anti-CTLA-4 antibodies resulted in rejection of preestablished tumors and that the mice lacking *Ctla4* gene (*Ctla4*^*-/-*^ mice) develop severe lymphoproliferative and lethal autoimmune phenotype [23–25].

Further studies showed that CTLA-4 engagement activated intrinsic signaling cascades in T-cells. CTLA-4 activation was reported to inhibit IL-2 production and T cell proliferation and induce cell cycle arrest through cross-talks with pathways regulating cell survival and proliferation, including PI3K, NFκB and MAPK pathways [26–30]. Based on the potential of CTLA-4 blockade for treatment of cancer seen in murine tumor models, anti-CTLA-4 antibodies were developed [24]. Among them, ipilimumab was approved for unresectable metastatic melanoma as well as adjuvant to surgery for ‘high-risk’ melanoma [31–38].

### PD-1

PD-1 (PDCD1 and CD279) is a cell surface receptor commonly seen on T cells, B cells and NK cells. Professor Honjo and coworkers are credited for the discovery of PD-1 through their studies on pathways of programmed cell death [39]. The details of human PD-1 gene location and the encoded protein are listed in Table 2. There is some similarity (21-33%) between extracellular domain of PD-1 and CTLA-4, but unlike CTLA-4, a dimeric protein, PD-1 lacks the extracellular cysteine residue required for covalent dimerization and exists as a monomer on cell surface and also in solution [40]. Basal level of PD-1 is seen on B cells but not on naïve T cells; its expression is induced upon activation of TCR/BCR. Apart from T cells, NK cells and B cells, PD-1 is also expressed on Tregs, NKT cells, activated monocytes and myeloid DCs. The ligands for PD-1, PD-L1 (B7-H1) and PD-L2 (B7-DC) are commonly expressed on macrophages and DCs [41, 42]. PD-L1 is also expressed on T-cells, B-cells, vascular endothelial cells, fibroblastic reticular cells, epithelial cells, pancreatic islet cells, astrocytes, neurons as well as on sites of immune privilege such as trophoblasts in placenta and retinal pigment epithelial cells [42–44]. Upon binding with their ligands, PD-1 receptors inhibit cell proliferation, cytokine secretion and cytotoxic ability of effector immune cells and thereby blunt the immune response [45]. Recently, using knock-in mice researchers from Tokushima University, Japan, showed that the function of PD-1 receptors was restricted during early stages of T-cell activation by cis interaction of CD80 and PD-L1 on APCs thereby preventing PD-L1/PD-1 binding [46].

PD-1 receptors are known to activate downstream signaling pathways and promote the differentiation of induced Treg (iTreg) cells in murine models through induction of FoxP3 expression [41]. Activation of PD-1 receptors was shown to result in phosphorylation of the tyrosine residue located within ITSM motifs of the cytoplasmic tails, recruitment of phosphatases SHP1 and SHP2 and dephosphorylation of downstream effectors such as Syk, PI3K, ZAP70 and CD3ζ. Through inhibition of PI3K pathway, PD-1 signaling was shown to prevent activation of the cell survival factor Bcl-xL and abrogate the expression of transcription factors that regulate the effector functions of T-cells such as GATA-3, T-bet and Eomes [44]. Interestingly, activation of TCR via CD28 or activation of downstream mediators of PI3K/Akt pathway such as STAT5 by cytokines including IL-2, IL-7 and IL-15 was shown to blunt the extent of PD-1 mediated inhibition [47, 48].

Early studies in knock out mice demonstrated the importance of PD-1 in regulation of immune response. While the phenotype was comparatively mild, mice lacking PD-1 developed autoimmune disorders such as lupus like syndrome, characterized by glomerulonephritis and arthritis and autoimmune dilated cardiomyopathy [49, 50]. PD-1/PD-L1 pathway is found to play a key role in escape of cancer from immunosurveillance, with PD-1 expression seen on effector T-cells and exhausted T-cells in tumor microenvironment (TME) and PD-L1 expression seen on cell surface in several types of cancers including bladder, lung, colon, breast, kidney, ovary, cervix, melanoma, glioblastoma, multiple myeloma and T-cell lymphoma [41, 43]. Blockade of PD-1/PD-L1 pathway to stimulate anti-tumor immune responses has been the most successful strategy to date. Three monoclonal anti PD-1antibodies, pembrolizumab, nivolumab and cemiplimab and 3 monoclonal anti-PD-L1 antibodies, atezolizumab, avelumab and durvalumab are approved by US FDA for the treatment of different types of cancer [9, 51–81].

## Rationale for combination

**Fig. 1:**
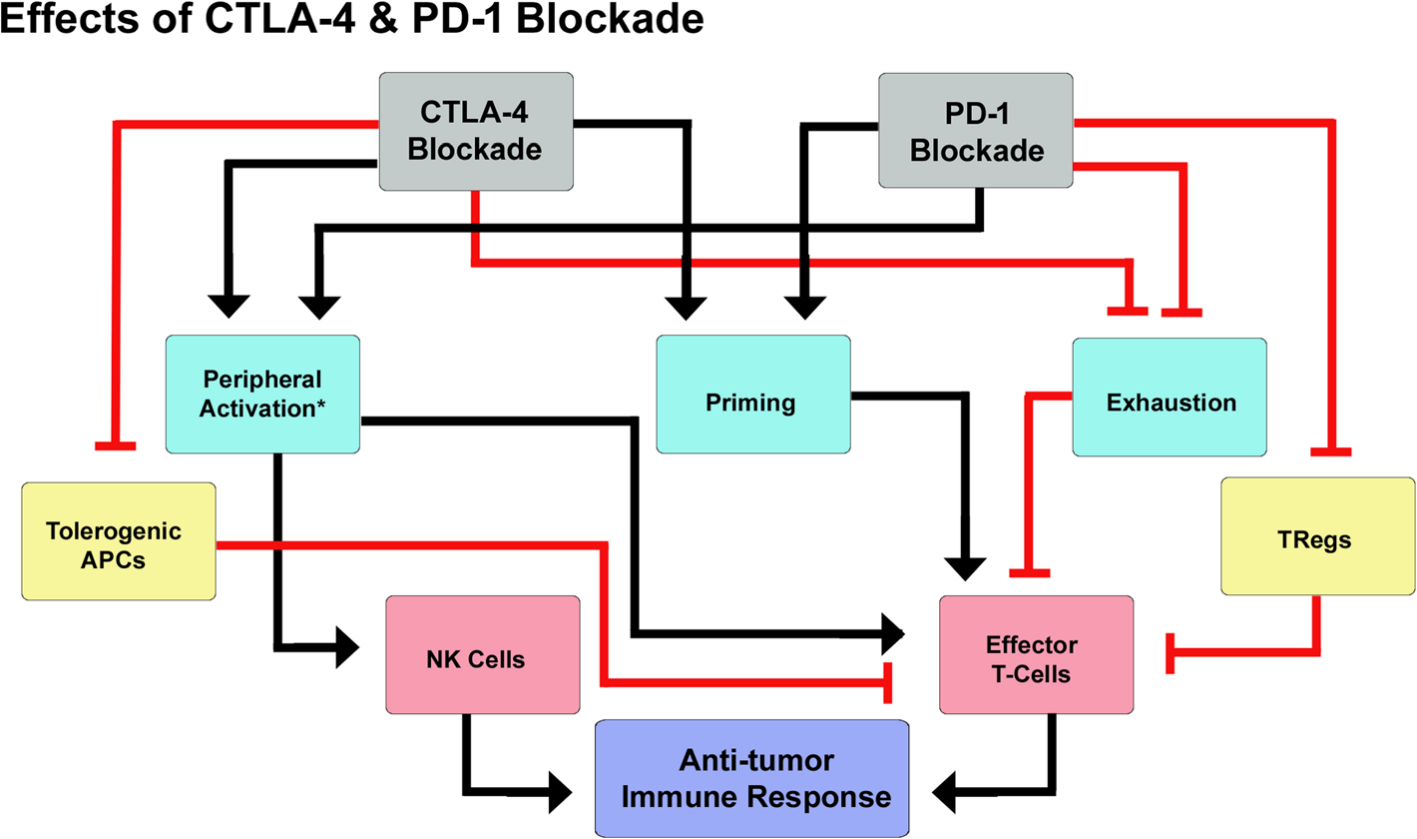
Effects of combined blockade of CTLA-4 and PD-1. *-NK cells do not express CTLA-4 and are not expected to be activated by CTLA-4 blockade

When administered as monotherapy in clinical studies, CTLA-4 and PD-1 blockers demonstrated impressive durable response rates, increased the survival time of responding patients significantly and had a manageable safety profile [4, 13–15]. However, benefits of monotherapy were limited by low response rates and only a fraction of patients were found to respond to the therapy [13]. For example, more than 50% of metastatic melanoma patients failed to respond to monotherapy as seen by objective response rates (ORR) for ipilimumab (10-16%) and for nivolumab and pembrolizumab (30-40%) [36, 37, 51, 52, 68, 69]. Combination of CTLA-4 and PD-1 blockade was thus proposed to increase the response rates and survival rates of the patients. It was thought that blockade of CTLA-4, which is primarily involved in regulation of T-cell activation in lymph nodes/tissues and in suppression of DC activity via Treg cells, would act synergistically with blockade of PD-1 that is mainly involved in inhibition of effector T-cell and NK cell activation in peripheral tissues and in induction of Treg cell differentiation (Fig. 1) [25, 49, 50, 82, 83]. Results from clinical trials that evaluated the efficacy of CTLA-4 plus PD-1 blockers and demonstrated the benefits of combination therapy are discussed in the following section.

## Clinical evidence

### Melanoma

Anti-CTLA-4 (ipilimumab) and anti-PD-1 (nivolumab and pembrolizumab) combination was studied extensively in metastatic melanoma patients and the efficacy of the combination was demonstrated in multiple clinical trials [84–94]. In a phase 1 study, ipilimumab plus nivolumab combination was reported to increase the ORR to 61% (*n*=44/72), with complete responses seen in 22% (*n*=16/72) patients. Patients assigned to combination therapy in the study reportedly had significantly lower incidence of disease progression or death; hazard ratio (HR) for disease progression or death in combination therapy group versus ipilimumab monotherapy was 0.40 (*p*<0.001) [85]. In another phase 2 study, patients treated with combination therapy increased the 2-year overall survival (OS) rate to 63.8% at the time of median follow-up time [92]. In the phase 3 study, patients treated with nivolumab plus ipilimumab had higher ORR (57%, 19% and 44% respectively), longer median progression free survival (PFS, 11.5, 2.9 and 6.9 months respectively) and lower incidence of disease progression or death (HR, 0.42 and 0.57 respectively, *p*<0.001 for both) compared to ipilimumab and nivolumab monotherapy [86]. Results from analyses of outcomes after 3-year and 4-year follow-up of the patients in the study further showed the superior benefits of combination therapy over monotherapy [87, 88]. Combination therapy showed sustained OS rate of over 50% at both 3-year and 4-year assessment (Table 3). Pooled analysis of data from patients treated with nivolumab alone or in combination with ipilimumab in clinical studies including phase 3 trials, further showed that patients receiving combination therapy had higher median PFS, 11.7 months for cutaneous melanoma patients and 5.9 months for mucosal melanoma patients compared to nivolumab monotherapy group (6.2 months and 3.0 months respectively) [95]. To address the increased incidence of adverse events seen with combination therapy, alterations in the sequence of administration of nivolumab and ipilimumab was tested in a phase 2 study, in which, patients either received nivolumab for six doses followed by a planned switch to ipilimumab for four doses or ipilimumab for four doses followed by nivolumab for six doses. Interestingly, disease progression was lower and overall survival was better when nivolumab was administered first followed by ipilimumab, but there was no significant difference in frequencies of treatment related grade 3-5 adverse events between the two groups [91].

**Table 3:** Clinical studies that supported approval of the combination

Patients	Trial, ID	Follow-up	Outcomes	Reference
Advanced melanoma	Phase 1 NCT01024231	≥24 weeks	ORR, 53% Grade 3-4 AEs, 53%	Wolchok et al 2013
Previously untreated advanced melanoma	Phase 1 NCT01927419	≥11 months	In patients with BRAF-WT tumors ORR 61% Median PFS, not reached HR for disease progression or death, 0.40 Grade 3-4 AEs, 54%	Postow et al 2015
Previously untreated advanced melanoma	Phase 3 NCT01844505	> 12 months	Median PFS, 11.5 months HR for death or disease progression, 0.42 Investigator assessed ORR, 57% Grade 3-4 AEs, 55%	Larkin et al 2015
Previously untreated advanced melanoma	Phase 3 NCT01844505	≥ 36 months	Median OS, not reached 3-year OS rate, 58% HR for death, 0.55 Grade 3-4 AEs, 59%	Wolchok et al 2017
Previously untreated advanced melanoma	Phase 3 NCT01844505	≥ 48 months	Median OS, not reached 4-year OS rate, 54% ORR, 58% HR for death, 0.54 HR for progression-free survival, 0.42 Grade 3-4 AEs, 59%	Hodi et al 2018
Advanced melanoma patients with at least one brain metastasis	Phase 2 NCT02320058	≥ 6 months	Rate of intracranial clinical benefit, 57%; Rate of extracranial clinical benefit, 56% 9-month PFS (global) rate, 57%; 9-month OS rate, 83 12-month OS rate, 82% Grade 3-4 AEs, 55%	Tawbi et al 2018
Advanced melanoma	Phase 2 NCT01783938	≥ 15 months	ORR, 56% Median OS, not reached 1-year OS rate, 76% Grade 3-5 AEs, 50%	Weber et al 2018
Previously untreated advanced clear cell renal cell carcinoma	Phase 3 NCT02231749	> 17 months	ORR, 42% Median OS, not reached HR for death, 0.63 Median PFS, 11.6 months HR for disease progression, 0.82	Motzer et al 2018
Previously treated, MMR/MSI-H positive advanced colorectal cancer	Phase 2 NCT02060188	> 9 months	ORR, 55% Median PFS, not reached 12-month PFS rate, 71% Median OS, not reached 12-month OS rate, 85% Grade 3-4 AEs, 32%	Overman et al 2018

#### Pembrolizumab plus ipilimumab combination

In a phase 1b study, efficacy of regular dose pembrolizumab plus low dose ipilimumab combination was studied in metastatic melanoma patients. Interestingly, pembrolizumab and low-dose ipilimumab combination also showed comparable efficacy with ORR of 61%, 1-year PFS rate of 69% and 1-year OS rate of 89% but had lower incidence of grade 3-4 adverse events (46%) [89]. Results from analysis of ‘real-world’ outcomes showed that metastatic cutaneous melanoma patients treated with the combination of pembrolizumab and low-dose ipilimumab had an overall response rate of 38% and lower incidence of grade 3-4 adverse events (18%) [96].

#### Nivolumab plus ipilimumab for surgically resectable ‘high-risk’ melanoma

Anti-CTLA-4 and anti-PD-1 combination was also tested for the treatment of melanoma in adjuvant and neoadjuvant settings. In a feasibility study, patients with palpable stage III melanoma received either four doses of ipilimumab and nivolumab combination after surgery (adjuvant setting) or two doses of the combination before surgery and two doses after surgery (neoadjuvant setting). The study reported that neoadjuvant administration of ipilimumab and nivolumab combination induced pathological responses in 78% (*N*=7/9) patients and had comparatively higher expansion of tumor resident T-cell clones. At the time of reporting (median follow-up, 25.6 months), none of the patients had relapse of the disease. Authors concluded that while the neoadjuvant therapy was promising, further research was needed to reduce toxicity while preserving efficacy [97].

### Renal cell carcinoma

Combination of anti-CTLA-4 (ipilimumab) and anti-PD-1 (nivolumab) antibodies for the treatment of metastatic renal cell carcinoma was first tested in a phase 1 study and was followed up in a phase 3 study (Table 3) [98, 99]. Phase 1 study was designed to test multiple dose regimens of the combination. Results showed that while the ORR (40.4% for both arms) and 2-year OS rate (67.3% and 69.6% respectively) was not different between patients who received nivolumab 3 mg/kg plus ipilimumab 1 mg/kg (N3/I1 group) and nivolumab 1 mg/kg plus ipilimumab 3 mg/kg (N1/I3 group), treatment-related grade 3-4 adverse events were comparatively higher in N1/I3 group (38.3% and 61.7% respectively) [98]. In the randomized phase 3 trial that followed, nivolumab 3 mg/kg plus ipilimumab 1 mg/kg was chosen for the treatment. The study reported 18-month OS rate of 75%, ORR of 42% (complete response rate, 9%) and median PFS of 11.6 months in the combination group. The incidence of death and disease progression or death in the combination group was lower compared to control (sunitinib) group (HR for death, 0.63, *p*<0.001, significant; HR for disease progression or death, 0.82, *p*=0.03, not significant per the prespecified 0.009 threshold) [99]. In a follow-up analysis, patient reported outcomes from the phase 3 trial were studied, which showed that patients in nivolumab plus ipilimumab group had fewer symptoms and had better health related quality of life compared to the control group [100].

### Colorectal cancer

Colorectal cancer with DNA mismatch repair-deficient (dMMR) or microsatellite instability high (MSI-H) positive tumors was expected to respond to immunotherapy due to high levels of tumor neoantigens, tumor-infiltrating lymphocytes and expression of immune checkpoints. In an open-label phase 2 study, blockade of PD-1 receptors with nivolumab recorded an ORR of 31%, disease control rate of 69% and 12-month OS rate of 73% [101]. In the follow-up report, investigators from the study showed that combination of nivolumab and ipilimumab had an investigator-assessed ORR of 55% and disease control rate of 80%. PFS rates at 9-month and 12-month were 76% and 71% respectively and OS rates were 87% and 85% respectively. Authors concluded that combination of nivolumab and ipilimumab had comparatively better efficacy and was a promising new treatment option for metastatic colorectal cancer patients with dMMR/MSI-H positive tumors [102].

### Lung cancer

#### Durvalumab plus tremelimumab for non-small cell lung cancer (NSCLC)

Multiple studies investigated the efficacy of anti-PD-1/PD-L1 plus anti-CTLA-4 antibodies in lung cancer (Table 4). The first study (phase 1b) evaluated the safety and efficacy of durvalumab (anti-PD-L1) and tremelimumab (anti-CTLA-4) combination in patients with advanced squamous or non-squamous NSCLC across five cancer centers in USA. The study reported clinical activity in patients with PD-L1 positive tumors as well as PD-L1 negative tumors with investigator assessed confirmed ORR in 23% patients [103].

**Table 4:** Clinical studies in Lung Cancer

Patients	Trial, ID	Follow-up	Outcomes	Reference
Advanced NSCLC	Phase 1b NCT02000947	24 weeks	ORR, 23% Grade 3-4 AEs, 35%	Antonia et al 2016
Treatment relapsed advanced SCLC	Phase 1/2 NCT01928394	≥ 12 weeks	ORR, 23% Median OS, 7.7 months 1-year OS rate, 43% Median PFS, 2.6 months 1-year PFS rate, 19% Grade 3-4 AEs, 30%	Antonia et al 2016
Untreated advanced NSCLC	Phase 1 NCT01454102	> 9 months	ORR, 47% Median PFS, 8.1 months 24-week PFS rate, 68% Grade 3-4 AEs, 37%	Hellman et al 2017
Untreated advanced NSCLC	Phase 2 NCT02659059	≥ 6 months	In patients with TMB≥10 mutations/megabase ORR, 44% Median PFS, 7.1 months 6-month PFS rate, 55% Grade 3-4 AEs, 29% (all patients)	Ready et al 2019
Untreated advanced NSCLC	Phase 3 NCT02477826	> 11 months	In patients with TMB≥10 mutations/megabase ORR, 45% Median PFS, 7.2 months 12-month PFS rate, 43% HR for disease progression or death, 0.58 Grade 3-4 AEs, 31%	Hellman et al 2018

#### Nivolumab plus ipilimumab for NSCLC

Safety and activity of nivolumab and ipilimumab combination as first-line therapy for NSCLC was tested in a phase 1 study. Two different dosage regimens of the combination including, nivolumab every 2 weeks plus ipilimumab every 12 weeks and nivolumab every 2 weeks plus ipilimumab every 6 weeks were evaluated in the study. At the time of reporting, confirmed ORR appeared to be slightly higher (47% versus 38% respectively) in patients receiving ipilimumab every 12 weeks compared to patients receiving ipilimumab every 6 weeks [104]. An open-label phase 3 trial was then initiated in patients with stage IV or recurrent NSCLC that was not previously treated with chemotherapy. The study showed that in patients with high tumor mutational burden (≥10 mutations per megabase) nivolumab plus ipilimumab combination achieved ORR of 45.3%, 1-year progression free survival rate of 42.6% and median PFS of 7.2 months. The relative incidence of disease progression or death was significantly lower in nivolumab plus ipilimumab combination group compared to chemotherapy group (HR for disease progression or death, 0.58, *p*<0.001). In patients with tumor mutational burden of at least 10 mutations per megabase and PD-L1 expression of at least 1%, nivolumab monotherapy group in the study had lower median PFS (4.1 months) compared to nivolumab plus ipilimumab combination (7.1 months); HR for disease progression or death between combination group and monotherapy group was 0.75 [105]. In the following open-label phase 2 study, the efficacy and safety of nivolumab plus ‘low-dose’ ipilimumab as first-line treatment for metastatic NSCLC was tested and the association of efficacy with PD-L1 expression and tumor mutational burden was assessed. Study showed that ORR was higher in patients with tumor mutational burden of at least 10 mutations per megabase and was not dependent on PD-L1 expression (48% in PD-L1≥1% group and 47% in PD-L1≤1% group), and proposed ≥10 mutations per megabase as the cutoff for tumor mutational burden [106].

#### Nivolumab plus ipilimumab for small cell lung cancer (SCLC)

In addition to NSCLC, combination of nivolumab and ipilimumab was tested in patients with advanced SCLC. In a multicenter phase 1/2 study, patients who relapsed after at least one previous platinum-containing regimen were treated with nivolumab plus ipilimumab or nivolumab alone. At the time of assessment, patients receiving combination of nivolumab and ipilimumab had higher ORR (23% versus 10%) and longer survival (median OS, 7.7 versus 4.4 months and 1-year OS rate, 43% versus 33%) compared to nivolumab monotherapy, further confirming the benefits of combining PD-1 and CTLA-4 blockers [107].

### Mesothelioma

Combination of anti-PD-1 and anti-CTLA-4 antibodies was tested in two phase 2 trials in patients with malignant pleural mesothelioma (Table 5). In the first study, a prospective single center, single arm trial, malignant pleural mesothelioma patients who progressed after at least one line of platinum-containing chemotherapy, were treated with nivolumab plus ipilimumab combination. The study noted that in the eligible patients with evaluable response, stable disease was achieved in 38% patients, partial response in 29% patients and disease control in 68% patients [108]. In the second study, a prospective, randomized, non-comparative, open label, multicenter trial, patients progressing after first-line or second-line pemetrexed or platinum-based treatments were treated with nivolumab plus ipilimumab combination or nivolumab alone. The study reported that in the intention-to-treat population, disease control was achieved in 52% patients in combination group and 40% patients in monotherapy group [109]. Authors from both studies concluded that nivolumab and ipilimumab combination showed promising activity in malignant pleural mesothelioma patients who progressed after chemotherapy and recommended confirming the efficacy in larger trials.

**Table 5:** Clinical studies in other cancer types

Cancer type	Patients	Trial, ID	Follow-up	Outcomes	Reference
Malignant pleural mesothelioma	Previously treated	Phase 2 NCT03048474	> 12 months	ORR, 38% Median PFS, 6.2 months 6-month PFS rate, 50% Median OS, not reached 12-month OS rate, 64% Grade 3-4 AEs, 38%	Disselhorst et al 2019
Malignant pleural mesothelioma	Previously treated	Phase 2 NCT02716272	> 16 months	ORR, 28% Median PFS, 5.6 months 12-month PFS rate, 23% Median OS rate, 15.9 months 12-month OS rate, 58% Grade 3-4 AEs, 26%	Scherpereel et al 2019
Unresectable Sarcoma	Previously treated	Phase 2 NCT02500797	> 12 months	Confirmed response, 16% Median PFS, 4.1 months Median OS, 14.3 months Grade 3-4 AEs, 14%	D’Angelo et al 2018
Esophagogastric cancer	Previously treated	Phase 1/2 NCT01928394		Investigator assessed ORR, 24% Median PFS, 1.4 months 12-month PFS rate, 17% Median OS, 6.9 months 18-month OS rate, 28% Grade 3-4 AEs, 35%	Janjigian et al 2018
Prostate cancer	Previously treated, AR-V7 positive	Phase 2 NCT02601014	≥ 1.9 months	ORR, 25% Median PFS,3.7 months Median OS, 8.2 months Grade 3-4 AEs, 46%	Boudadi et al 2018

### Esophagogastric cancer

Benefits of combined blockade of PD-1 and CTLA-4 was evaluated in a multicenter trial in patients with locally advanced or metastatic esophagogastric cancers (Table 5). Patients who relapsed after prior chemotherapy received either nivolumab monotherapy or nivolumab plus ipilimumab combination in the study. Analysis of the outcomes revealed that investigator-assessed ORR were seen in 24% patients receiving the combination of nivolumab and ipilimumab and in 12% receiving nivolumab alone. 12-month PFS rates 17% and 8%, and 12-month OS rates were 35% and 39% respectively. Interestingly, out of the two different dose cohorts included to evaluate the combination, patients receiving nivolumab 1 mg/kg and ipilimumab 3 mg/kg had comparatively better objective response rate (24% versus 8% respectively), 12-month PFS rate (17% versus 10% respectively) and 12- month OS rate (35% versus 24%). Authors noted that phase 3 studies testing the efficacy of combination in earlier lines of therapy for esophagogstric cancer were ongoing [110].

### Prostate Cancer

Efficacy of anti-PD-1 and anti-CTLA-4 antibodies in metastatic prostate cancer patients was tested in a single center prospective phase 2 trial (Table 5). In the study, patients with androgen receptor variant 7 (AR-V7) positive tumors were treated with nivolumab plus ipilimumab combination. At the time of report, ORR in patients with measurable disease was 25%, median PFS was 3.7 months and OS was 8.2 months. Authors observed that outcomes appeared to be better in tumors with DNA repair deficiency (DRD positive tumors) compared to DRD negative tumors (ORR, 40% vs 0% respectively; HR for disease progression, 0.31 and HR for death, 0.41) and concluded that further studies in larger cohort were needed to validate the efficacy of the combination [111].

### Sarcoma

Safety and activity of PD-1 blockade alone or in combination with CTLA-4 blockade was evaluated in an open-label, non-comparative, randomized phase 2 study in sarcoma patients who received at least one previous line of systemic therapy (Table 5). Patients enrolled in the study received either nivolumab alone or combination of nivolumab and ipilimumab. At the time of assessment, nivolumab and ipilimumab combination group had comparatively higher confirmed responses (16% versus 5%), longer median PFS (4.1 months versus 1.7 months) and longer median OS (14.3 months versus 10.7 months). Authors concluded that nivolumab monotherapy showed limited efficacy in sarcoma patients and did not warrant further study, whereas nivolumab and ipilimumab combination showed promising efficacy and needed further confirmation through larger randomized study [112].

## Summary

As hypothesized based on their mechanism of action, combination of PD-1 and CTLA-4 blockers has been successful in increasing the response rates and median survival time in cancer patients. Nivolumab plus ipilimumab combination has been approved for 3 indications including, metastatic melanoma, advanced renal cell carcinoma and colorectal cancer with MMR and MSI-H aberrations. Multiple studies demonstrated increased response rates and survival rates in lung cancer patients treated with combination of nivolumab and ipilimumab, and the combination was also seen to be effective in difficult to treat types of cancers such as mesothelioma and sarcoma. However, majority of the studies tested the combination of nivolumab and ipilimumab and only handful of studies evaluated the combination of other PD-1/PD-L1 and CTLA-4 blockers. Further studies may be needed to confirm the efficacy of combining other PD-1 blockers such as pembrolizumab and cemiplimab or PD-L1 blockers such as atezolizumab, avelumab and durvalumab with CTLA-4 blockers such as ipilimumab or tremelimumab. Furthermore, combining nivolumab and ipilimumab was shown to increase the incidence of adverse events and to precipitate auto immunity [113–115]. The severity and incidence of adverse events was shown to be mitigated partly by changing the dose, changing the regimen and changing the sequence of administration of the drugs [89, 91]. Interestingly, the dose of nivolumab and ipilimumab that showed promising efficacy and limited toxicity appeared to vary with cancer type. For instance, 1 mg/kg nivolumab plus 3 mg/kg ipilimumab every 3 weeks was effective dose for treatment of metastatic melanoma and esophagogastric cancer, whereas 3 mg/kg nivolumab plus 1 mg/kg ipilimumab every 3 weeks was effective dose for metastatic renal cell carcinoma, metastatic colorectal cancer and sarcoma [86, 100, 102, 112]. Similarly, for non-small cell lung cancer, nivolumab 3 mg/kg every 2 weeks plus ipilimumab 1 mg/kg every 6-12 weeks was shown to be the effective combination. The differences in effective doses of PD-1 and CTLA-4 blockers in the combination point to the complex differences in tumor microenvironment in various cancer sub-types. Additional studies are ongoing to titrate the dose, regimen and the administration sequence of the combination (Additional file 1: Table S1 and Additional file 2: Table S2). The results from the studies could provide additional insights into immunosuppressive mechanisms in TME and the significance of CTLA-4 plus PD-1 blockade in respective types of cancer, and help in identifying the combination dose with desired efficacy and adverse event profile.

## Conclusions

In conclusion, combination of CTLA-4 and PD-1 blockers was effective in increasing the response and survival rates in multiple cancer types, but it also increased the incidence of adverse events. Further studies may be needed to reduce the incidence and intensity of the adverse events while preserving the efficacy of the combination. Additional studies are also needed to confirm the efficacy of combination of other CTLA-4 (tremelimumab) and PD-1/PD-L1 (pembrolizumab, cemiplimab, atezolizumab, durvalumab, and avelumab) blockers.

## Additional files

Additional file 1: **Table S1.** Ongoing clinical trials testing ipilimumab and nivolumab combination. (DOCX 26 kb)

Additional file 2: **Table S2.** Ongoing trials testing ipilimumab and pembrolizumab or tremilimumab and durvalumab combination. (DOCX 18 kb)

## Data Availability

All data generated or analysed during this study are included in this published article [and its additional files]

## Abbreviations

APC: Antigen presenting cell
ARv: Androgen receptor variant
CD: Cluster of differentiation
CTLA-4: Cytotoxic T-lymphocyte associated protein 4
dMMR: Mismatch repair deficient
DRD: DNA repair deficiency
HR: Hazard ratio
IL-2: Interleukin-2
MAPK: Mitogen-activated protein kinase
MSI-h: Microsatellite instability high
NK cells: Natural killer cells
NKT cells: Natural killer T-cells
NSCLC: Non-small cell lung cancer
ORR: Objective response rate
OS: Overall survival
PD-1: Programmed cell death protein 1
PFS: Progression free survival
PI3K: Phosphoinositide 3-kinase
SCLC: Small cell lung cancer
TME: Tumor microenvironment
Tregs: Regulatory T-cells
