# Supplementary material for: Combination of CTLA-4 and PD-1 blockers for treatment of cancer
Source: J Exp Clin Cancer Res. 2019 Jun 13;38:255. doi: 10.1186/s13046-019-1259-z (PMC6567914; doi:10.1186/s13046-019-1259-z)
Supplement: Supplementary file 1 — Table S1. Ongoing clinical trials testing ipilimumab and nivolumab combination. (DOCX 26 kb) [file 13046_2019_1259_MOESM1_ESM.docx]

**Additional file 1: Table S1.** Ongoing clinical trials testing ipilimumab and nivolumab combination

| **Multiple Cancer Types** | | |
| --- | --- | --- |
| **Phase** | **Trial ID** | **Status** |
| 1,2 | NCT03241173 | Active, not recruiting |
| 1,2 | NCT01928392 | Active, not recruiting |
| 2 | NCT03668119 | Recruiting |
| 2 | NCT03130950 | Recruiting |
| 1,2 | NCT03126110 | Recruiting |
| 2 | NCT03651271 | Recruiting |
| 1,2 | NCT02983045 | Recruiting |
| 1 | NCT03195478 | Recruiting |
| 2 | NCT03461952 | Recruiting |
| 1 | NCT024088861 | Recruiting |
| 2 | NCT02553642 | Recruiting |
| 2 | NCT03342417 | Recruiting |
| 1,2 | NCT03326258 | Withdrawn |
| **Gastrointestinal Cancers** | | |
| **Phase** | **Trial ID** | **Status** |
| 2 | NCT03604991 | Not yet recruiting |
| 2 | NCT03443856 | Not yet recruiting |
| 1,2 | NCT03682276 | Not yet recruiting |
| 2 | NCT03510871 | Not yet recruiting |
| 2 | NCT03416244 | Recruiting |
| 2 | NCT02880020 | Recruiting |
| 2 | NCT02880020 | Recruiting |
| 2 | NCT03350126 | Recruiting |
| 2 | NCT03350126 | Recruiting |
| 2 | NCT03693846 | Recruiting |
| 2 | NCT03146650 | Recruiting |
| 2 | NCT03172624 | Recruiting |
| 2 | NCT03222076 | Recruiting |
| **Lung Cancer** | | |
| **Phase** | **Trial ID** | **Status** |
| 2 | NCT02659059 | Active, not recruiting |
| 3 | NCT02538666 | Active, not recruiting |
| 2 | NCT03083691 | Enrolling by invitation |
| 2 | NCT03670056 | Not yet recruiting |
| 3 | NCT03351361 | Not yet recruiting |
| 2 | NCT02046733 | Recruiting |
| 2 | NCT03262779 | Recruiting |
| 2 | NCT03091491 | Recruiting |
| 2 | NCT03425331 | Recruiting |
| 2 | NCT03001882 | Recruiting |
| 2 | NCT02259621 | Recruiting |
| 2 | NCT02259621 | Recruiting |
| 4 | NCT02869789 | Recruiting |
| 2 | NCT03256136 | Recruiting |
| 3 | NCT02785952 | Recruiting |
| 3 | NCT03048136 | Withdrawn |
| **Melanoma** | | |
| **Phase** | **Trial ID** | **Status** |
| 3 | NCT01844505 | Completed (has results) |
| 2 | NCT01783938 | Completed (has results) |
| 3 | NCT02905266 | Active, not recruiting |
| 2 | NCT01585194 | Active, not recruiting |
| 1 | NCT02437279 | Active, not recruiting |
| 2 | NCT02970981 | Active, not recruiting |
| 2 | NCT02731729 | Active, not recruiting |
| 2 | NCT02626962 | Active, not recruiting |
| 3 | NCT02599402 | Active, not recruiting |
| 2 | NCT02320058 | Active, not recruiting |
| 2 | NCT02374242 | Active, not recruiting |
| 2 | NCT01927419 | Active, not recruiting |
| 3 | NCT02714218 | Active, not recruiting |
| 3 | NCT03068455 | Active, not recruiting |
| 2 | NCT02978443 | Recruiting |
| 2 | NCT03122522 | Recruiting |
| 1 | NCT03597282 | Recruiting |
| 2 | NCT03728465 | Recruiting |
| 2 | NCT03241186 | Recruiting |
| 2 | NCT03241186 | Recruiting |
| 2 | NCT03528408 | Recruiting |
| 1,2 | MCT02941744 | Recruiting |
| 2 | NCT02523313 | Recruiting |
| 1,2 | NCT02857569 | Recruiting |
| 2 | NCT03033576 | Recruiting |
| 2 | NCT03472586 | Suspended (Slow accural) |
| **Breast Cancer** | | |
| **Phase** | **Trial ID** | **Status** |
| 2 | NCT03789110 | Not yet recruiting |
| 2 | NCT03546686 | Not yet recruiting |
| 2 | NCT03650894 | Not yet recruiting |
| 1 | NCT02453620 | Recruiting |
| 2 | NCT03409198 | Recruiting |
| 2 | NCT02892734 | Recruiting |
| 2 | NCT02892734 | Recruting |
| **Renal Cell Carcinoma** | | |
| **Phase** | **Trial ID** | **Status** |
| 2 | NCT03029780 | Active, not recruiting |
| 4 | NCT02982954 | Active, not recruiting |
| 2 | NCT03117309 | Recruiting |
| 2 | NCT03297593 | Recruiting |
| 3 | NCT03138512 | Recruiting |
| 2 | NCT03177239 | Recruiting |
| 2 | NCT03274258 | Recruiting |
| **Head and Neck Cancer** | | |
| **Phase** | **Trial ID** | **Status** |
| 2 | NCT03406247 | Not yet recruiting |
| 3 | NCT02741570 | Active, not recruiting |
| 2 | NCT02823574 | Active, not recruiting |
| 2 | NCT02919683 | Recruiting |
| **Prostate Cancer** | | |
| **Phase** | **Trial ID** | **Status** |
| 2 | NCT02985957 | Active, not recruiting |
| 2 | NCT03570619 | Recruiting |
| 2 | NCT03061539 | Recruiting |
| **Ovarian Cancer** | | |
| **Phase** | **Trial ID** | **Status** |
| 2 | NCT02498600 | Active, not recruiting |
| 2 | NCT03333616 | Recruiting |
| 1 | NCT03508570 | Recruiting |
| 1 | NCT03508570 | Recruiting |
| **Sarcoma** | | |
| **Phase** | **Trial ID** | **Status** |
| 1,2 | NCT02304458 | Recruiting |
| 2 | NCT03219671 | Recruiting |
| 2 | NCT02428192 | Completed |
| **Hematological malignancies** | | |
| **Phase** | **Trial ID** | **Status** |
| 1 | NCT02846376 | Recruiting |
| 1 | NCT03600155 | Recruiting |
| 1,2 | NCT02681302 | Recruiting |
| **Others** | | |
| **Phase** | **Trial ID** | **Status** |
| 2 | NCT03048474 | Active, not recruiting |
| 2 | NCT02716272 | Active, not recruiting |
| 2 | NCT03097939 | Recruiting |
| 2 | Nct02834013 | Recruiting |
| 2 | NCT03420521 | Recruiting |
| 2 | NCT03246958 | Recruiting |
| 1 | NCT03233152 | Recruiting |
| 2 | NCT02939300 | Recruiting |
| 2 | NCT03521830 | Recruiting |
| 2 | NCT02314169 | Recruiting |
| 2 | NCT03520491 | Recruiting |
| 1 | NCT03387761 | Recruiting |
