# Supplementary material for: Combination of CTLA-4 and PD-1 blockers for treatment of cancer
Source: J Exp Clin Cancer Res. 2019 Jun 13;38:255. doi: 10.1186/s13046-019-1259-z (PMC6567914; doi:10.1186/s13046-019-1259-z)
Supplement: Supplementary file 2 — Table S2. Ongoing trials testing ipilimumab and pembrolizumab or tremilimumab and durvalumab combination. (DOCX 18 kb) [file 13046_2019_1259_MOESM2_ESM.docx]

**Additional file 2: Table S2.** Ongoing trials testing ipilimumab and pembrolizumab or tremilimumab and durvalumab combination

| **Multiple Cancer Types** | | |
| --- | --- | --- |
| **Phase** | **Trial ID** | **Status** |
| 1, 2 | NCT02978482 | Recruiting |
| 2 | NCT02879162 | Recruiting |
| 2 | NCT02938793 | Recruiting |
| 2 | NCT03158064 | Recruiting |
| 2 | NCT03081923 | Recruiting |
| **Genitourinary Cancers** | | |
| **Phase** | **Trial ID** | **Status** |
| 2 | NCT03026062 | Recruiting |
| 2 | NCT03234153 | Recruiting |
| 2 | NCT03430895 | Recruiting |
| 3 | NCT03288532 | Recruiting |
| 1 | NCT02762006 | Recruiting |
| 2 | NCT02788773 | Recruiting |
| 2 | NCT03204812 | Recruiting |
| Early phase 1 | NCT03132467 | Recruiting |
| 2 | NCT03608865 | Recruiting |
| 2 | NCT03015129 | Recruiting |
| **Lung Cancer** | | |
| **Phase** | **Trial ID** | **Status** |
| 2 | NCT03319316 | not yet recruiting |
| 3 | NCT03703297 | Recruiting |
| 3 | NCT03302234 | Recruiting |
| 2 | NCT03130764 | Withdrawn (PI Transferred) |
| **Others** | | |
| **Phase** | **Trial ID** | **Status** |
| 2 | NCT03753919 | not yet recruiting |
| 2 | NCT02743819 | Recruiting |
| 1,2 | NCT03784066 | Recruiting |
| 2 | NCT03022500 | Recruiting |
| 2 | NCT02815995 | Recruiting |
| 2 | NCT03095274 | Recruiting |
| 2 | NCT02558894 | Completed |
